# Navigating diagnostic complexity: A case report on uterine lipoleiomyoma, unveiling its benign nature amidst characteristics resembling liposarcoma

**DOI:** 10.18632/oncoscience.621

**Published:** 2025-07-02

**Authors:** Seetu Palo, Mishu Mangla, Annapurna Srirambhatla, Anwesha Dutta Chowdhury, Naina Kumar

**Affiliations:** ^1^Department of Pathology and Laboratory Medicine, All India Institute of Medical Sciences, Bibinagar 508126, Hyderabad, Telangana, India; ^2^Department of Obstetrics and Gynaecology, All India Institute of Medical Sciences, Bibinagar 508126, Hyderabad, Telangana, India; ^3^Department of Radiodiagnosis, All India Institute of Medical Sciences, Bibinagar 508126, Hyderabad, Telangana, India

**Keywords:** lipoleiomyoma, variant, leiomyoma, post-menopausal, case report

## Abstract

Lipoleiomyomas, rare variants of uterine leiomyomas, are characterized by the presence of mature adipocytes along with benign smooth muscle cells. The literature on this is limited to a few case reports and observational studies only. Presented here is a rare case of co-existing intramural and subserosal uterine lipoleiomyoma in a post-menopausal woman who had attained menopause 25 years prior. The 75-year-old patient, with a history of hypertension and diabetes, presented with lower abdominal pain. Imaging revealed an intramural degenerated fibroid in the anterior wall, measuring 7 × 6 × 5 cm, and another subserosal fibroid, measuring 2.5 × 2 × 1.5 cm, in the posterior uterine wall. A total abdominal hysterectomy with bilateral salpingo-oophorectomy was performed, with microscopy revealing lipoleiomyoma with high intra-tumoral mast cells and eosinophils. This case highlights that lipoleiomyomas can present many years after attaining menopause. Diligent microscopic examination should be carried out to render an accurate diagnosis and to rule out other adipocyte-containing neoplastic lesions. This uncommon variant of uterine lipoleiomyoma poses a distinctive set of considerations for healthcare professionals, and our report seeks to contribute to the expanding knowledge base surrounding this unique condition.

## INTRODUCTION

**Figure 1:**
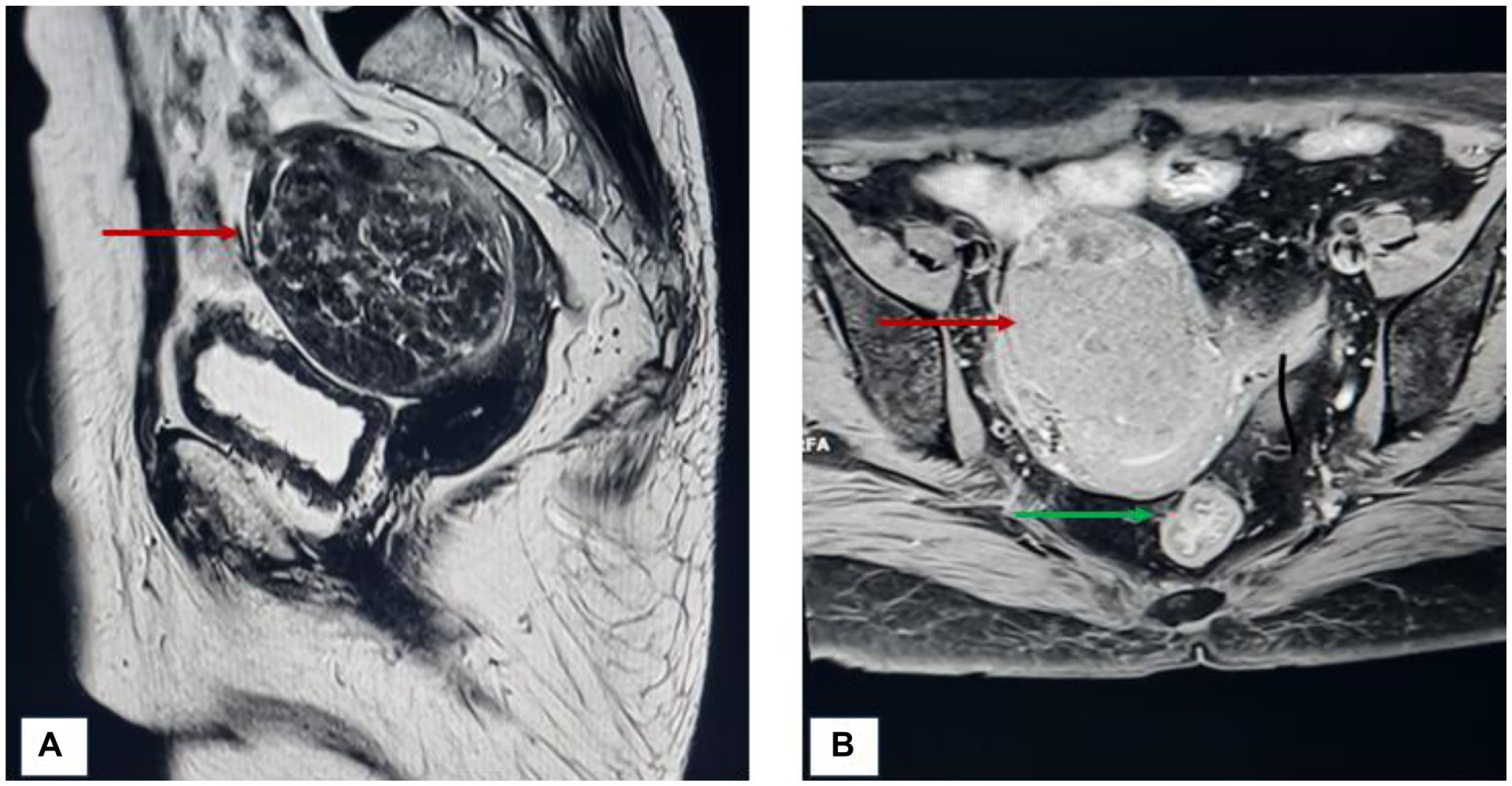
(**A**) T1 weighted (sagittal plane) image showing fibroid (red arrow) in the anterior uterine wall. (**B**) T2 weighted (transverse plane) image showing fibroid in the anterior uterine wall along with another subserosal fibroid (green arrow) arising from posterior wall.

A 75-year-old, post-menopausal woman, presented to the gynecology outpatient department with a complaint of dull aching, moderate pain of insidious onset, localized in the lower abdomen from the last 9 months. There was no history of post-menopausal or post-coital bleeding. There was no history of loss of appetite or weight, or bowel and bladder disturbances. The patient had attained menopause 25 years back and was not on any hormone replacement therapy. Her obstetric history revealed that she had four pregnancies, all of which were conceived spontaneously. She was on medication for hypertension and diabetes for the last 16 years. She had undergone stenting for triple vessel coronary artery disease 6 years back. The patient was moderately built with a body mass index (BMI) of 29.6. General and other systemic examinations were unremarkable. Lab investigations, including serum lipid profile and thyroid profile, were within normal limits. Per-abdominal examination revealed a soft, abdominopelvic mass of 14 weeks gravid uterus, with restricted mobility. Upon speculum examination, the cervix was flushed with the vagina and the vaginal walls appeared atrophic and pale. On bimanual examination, the uterus was normal in size, and the same soft mass of approximately 8 × 8 cm was palpated. These findings were reconfirmed on rectal examination as well. Magnetic Resonance Imaging (MRI) of the abdomen was performed which revealed an intramural fibroid in the anterior wall, measuring 70 × 65 mm, and a smaller subserosal fibroid in the posterior uterine wall (Figure 1).

**Figure 2:**
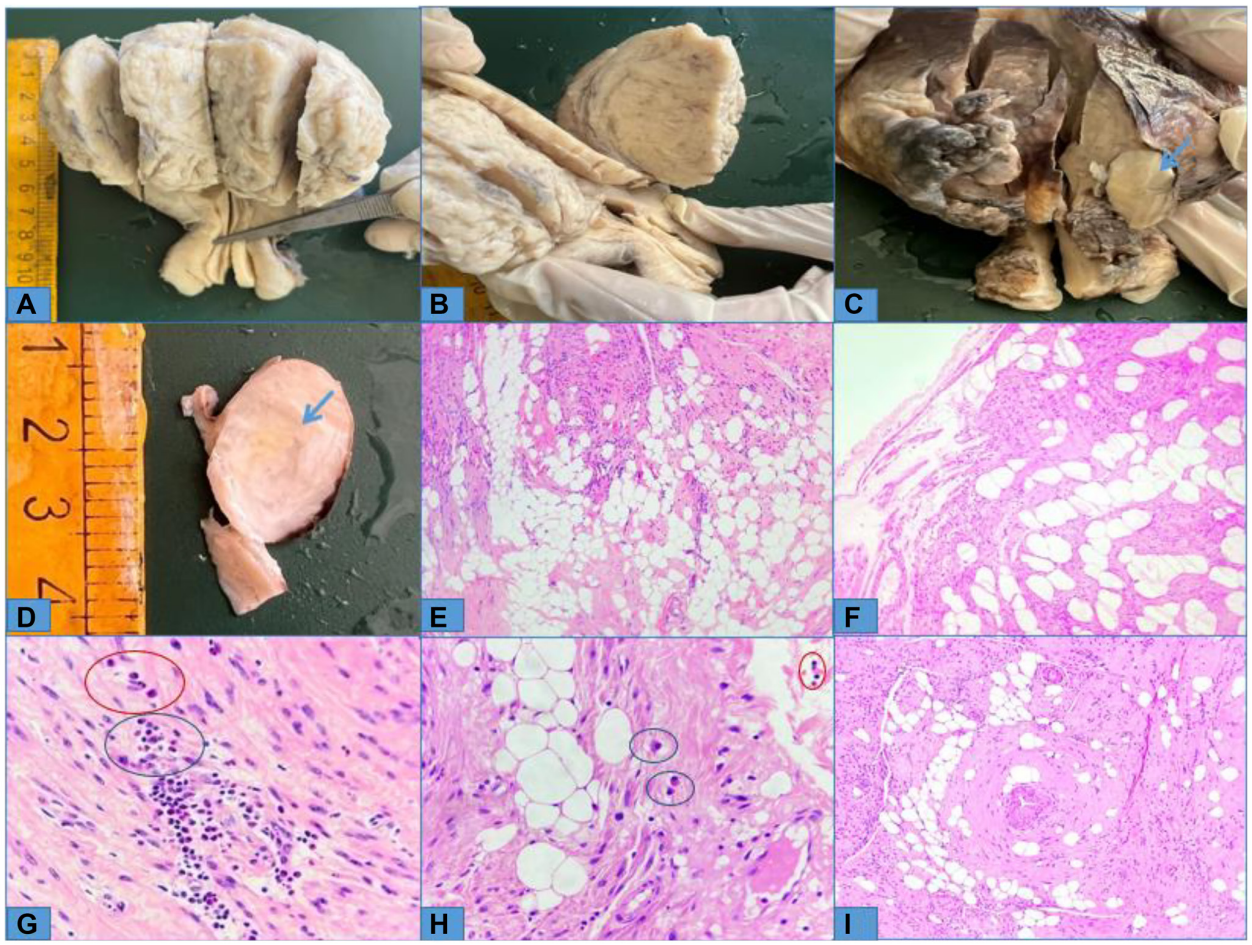
(**A**, **B**) Cut-surface of hysterectomy specimen displaying anterior uterine wall fibroid with no yellowish areas; (**C**, **D**) Cut-surface of subserosal fibroid showing yellowish area in the centre (blue arrow); (**E**, **F**) Microphotographs of intramural and subserosal fibroid, respectively, showing aggregates of mature adipocytes intermingled with bundles of smooth muscle cells (100×, Haematoxylin and Eosin); (**G**, **H**) Higher power showing intra-tumoral eosinophils (few circled in red) and mast cells (few circled in blue) (400×, Haematoxylin and Eosin); (**I**) A focus showing a vessel with a surrounding collar of smooth muscle cells and adipocytes (100×, Haematoxylin and Eosin).

A total abdominal hysterectomy with bilateral salpingo-oophorectomy was performed. Grossly, the uterus was enlarged to approximately 14 weeks, measuring 11 × 7 × 4 cm in size. Externally, a 2.5 × 2 × 1.5 cm subserosal fibroid was noted on the posterior uterine wall (Figure 2A, 2B), which showed fatty component. On sectioning the uterus, an intramural fibroid of 7 × 6 × 5 cm was noted involving the anterior wall (Figure 2C, 2D). However, no fatty component was detected grossly and it resembled conventional fibroid upon gross examination. Microscopically, both the masses showed features of lipoleiomyoma, comprising of interlacing bundles of benign smooth muscle cells intermingled with nests of mature adipocytes. The smooth muscle cells displayed oval to spindled, bland nuclei with blunt ends and variable amounts of eosinophilic cytoplasm (Figure 2E, 2F). Many mast cells and eosinophils were seen scattered within the tumor (Figure 2G, 2H). There was no cytologic atypia mitotic activity or tumor necrosis. Sections from the cervix showed features of chronic cervicitis and the endometrium showed atrophic changes. Bilateral adnexa were macro- and microscopically unremarkable. The post-operative period was uneventful.

## DISCUSSION

Lipoleiomyomas are rare histological variants of the commonly encountered uterine leiomyomas. Apart from interlacing bundles of benign smooth muscle cells, lipoleiomyoma contains variable proportions of mature adipocytes. Literature on lipoleiomyoma is scarce, limited to a few case reports and a handful of observational studies. Although benign, it is important to be aware of this entity as it can sometimes cause a diagnostic conundrum [1]. Here, we present a rare case of co-existing subserosal and intramural uterine lipoleiomyoma with high intra-tumoral mast cells and eosinophils in a post-menopausal woman, who attained menopause 25 years prior. Lipoleiomyomas account for about 0.03–2.1% of all uterine leiomyomas [2]. Contrary to conventional leiomyomas, these are relatively more common in perimenopausal or postmenopausal women [3]. In a series of 76 cases of lipoleiomyoma, 82.8% of cases were encountered in the postmenopausal age group, with a mean age of 55.49 years [3]. Of these, only nine (9/76; 11.8%) were subserosal in location and only six (6/76; 8.5%) women had two synchronously occurring lipoleiomyomas. In another clinicopathologic study of 50 cases of lipoleiomyomas, the mean age of presentation was 53.9 years (range: 29 to 92 years) and none of the patients had more than one lipoleiomyoma [2]. In yet another series of 17 lipoleiomyomas, only a single case had two lipoleiomyomas [4]. In our case, the women presented at the age of 75 years, much later than the usual documented age of presentation, and had two lipoleiomyomas, one being subserosal in location, which is also infrequently seen. Rare cases of extra-uterine lipoleiomyoma are also on record [5–8].

Regarding its etiopathogenesis, recent evidence suggests that inhibition of WNT/β-catenin signaling secondary to hypoxia leads to adipocytic metaplasia or trans-differentiation of uterine leiomyoma cells, resulting in the formation of lipoleiomyoma [9]. It is also proposed that uterine lipoleiomyoma may be associated with systemic diseases including hyperlipidemia, hypothyroidism, and diabetes mellitus [10]. It is also hypothesized that changes in lipid metabolism occurring during menopause might play a contributory role in the genesis of these tumors, and hence their commonality in post-menopausal women [1]. In our case, the patient was diabetic and had a BMI of 29.6 which corroborates this hypothesis.

Swanson et al., based on histomorphology, classified lipoleiomyoma into further sub-types, and called tumors showing >80% adipocytes as ‘adipocyte-rich lipoleiomyoma’ [8]. Akbulut et al. proposed a three-tier grading system depending upon the intra-tumoral fat content, according to which the current case can be labeled as ‘grade 2’ as there was a moderate number of adipocytes multifocally [3]. Although a significant number of adipocytes were seen microscopically, the intramural lesion did not show any foci of yellowish discoloration upon gross examination as expected. The fat component was not very evident upon MRI as well, likely due to tiny clusters of adipocytes rather than foci large enough to be detected upon imaging. Hence, our case highlights that imaging and gross appearance of these tumors can be deceptive and resemble that of conventional leiomyomas without macroscopic or radiologically detectable fat, which might lead to missing out or under-reporting of these cases if adequate sections of the lesion are not grossed and processed.

Microscopically, our case showed high intra-tumoral content of mast cells and eosinophils, similar to the findings of Wang et al. [2]. The presence of mast cells and eosinophils has been reported in conventional leiomyomas as well, but their true significance in pathogenesis and disease course remains enigmatic [11]. In addition, we also found a few thick-walled blood vessels interspersed (Figure 2I), as observed by Aung et al. [4], which can raise the suspicion of angiomyolipoma. Before labeling a case as lipoleiomyoma, its sinister histologic mimickers, such as angiomyolipoma and liposarcoma, should be ruled out by careful microscopic examination, especially in the set of elderly patients in whom the suspicion for aggressive malignancies remains high. Due attention should also be given to looking for areas of cytologic atypia, increased mitotic activity, and necrosis to rule out atypical leiomyoma, smooth muscle tumors of undetermined malignant potential, and leiomyosarcoma. Light microscopic examination is the gold standard for diagnosing lipoleiomyoma and immunohistochemistry has limited utility. Immunohistochemistry can only be of help in ruling out other differential diagnoses but is rarely required. There are few reports of lipoleiomyoma exhibiting an epithelioid smooth muscle component and/or bizarre nuclei but had an indolent clinical outcome [4, 8].

Although, lipoleiomyomas are completely benign, they may cause a diagnostic dilemma, with other malignant conditions [12]. The clinical differential diagnosis includes myxoid liposarcoma, angio-lipoleiomyoma, lipoma, and angiolipoma, all of which are known to manifest in the uterine corpus of postmenopausal women. However, key characteristics such as cellular pleomorphism, brisk mitotic activity, presence of lipoblasts, and infiltrative margins are typically absent in benign tumors. While lipoleiomyomas have been reported to occasionally display atypical smooth muscle cells and lipoblasts, they are generally distinct from myxoid liposarcomas. Additionally, other tumors to consider within this diagnostic realm include intravenous leiomyomatosis and endometrial stromal sarcoma, both of which may exhibit myxoid changes or adipose differentiation in rare cases. Inflammatory myofibroblastic tumor is another consideration, as it shares a myxoid background with myxoid liposarcoma. However, it typically consists of a benign spindle cell proliferation with a low mitotic rate and may exhibit infiltrative growth patterns resembling myxoid liposarcoma. Yet, distinguishing features such as fascicular growth of spindle cells with eosinophilic cytoplasm, along with the presence of a lymphoplasmacytic inflammatory infiltrate and anaplastic lymphoma kinase positivity, help set it apart. Lastly, while rare, gastrointestinal stromal tumors and nerve sheath tumors can also involve the uterus and thus merit consideration within this differential diagnosis [2, 12–17].

While the diagnosis in our case was confidently established through classical histomorphological features, we acknowledge that immunohistochemistry (IHC) can be a valuable adjunct in selected cases. Immunohistochemistry (IHC) serves as a valuable adjunct in challenging cases. Lipoleiomyomas typically express smooth muscle markers such as desmin and h-caldesmon in the smooth muscle component, and S100 protein in the adipocytic component [8]. Notably, they are negative for MDM2 and CDK4 [8, 18]. IHC may be particularly helpful in excluding histological mimics such as angiomyolipoma (HMB-45 positive), atypical lipomatous tumors (MDM2/CDK4 positivity), or other smooth muscle tumors with ambiguous features [18]. However, in our case, the absence of atypia, mitosis, necrosis, and the presence of characteristic intermingling of mature adipocytes with bland smooth muscle bundles rendered the diagnosis definitive without the need for IHC. Nonetheless, had there been any diagnostic ambiguity, IHC could have enhanced diagnostic accuracy.

To conclude, the possibility of a lipoleiomyoma variant has to be kept in mind in any post-menopausal women with uterine fibroids. It can come into clinical attention many years after attaining menopause. It can present as more than one lesion and can be of subserosal location as well. Grossly, lipoleiomyoma may not show yellowish discoloration, especially if the fat content is low. Adequate sampling and diligent microscopic examination should be carried out to render an accurate diagnosis to rule out other adipocyte-containing fatal neoplastic lesions and to exclude leiomyosarcoma. However, considering the single-case nature of the report, observations from this case should be interpreted with caution and cannot be extrapolated broadly without further supporting evidence from larger studies.
